# Stereotactic Lung Re-Irradiation After a First Course of Stereotactic Radiotherapy with In-Field Relapse: A Valuable Option to Be Considered

**DOI:** 10.3390/cancers17030366

**Published:** 2025-01-23

**Authors:** Assim Sahin, Edouard Romano, Alessio Casutt, Raphaël Moeckli, Véronique Vallet, Shaïma El Chammah, Mahmut Ozsahin, Rémy Kinj

**Affiliations:** 1 Faculty of Biology and Medicine, University of Lausanne (UNIL), 1011 Lausanne, Switzerland; 2 Department of Oncology, Radio-Oncology Service, Lausanne University Hospital, University of Lausanne (UNIL), 1011 Lausanne, Switzerland; 3 Division of Pulmonology, Department of Medicine, Lausanne University Hospital (CHUV), University of Lausanne (UNIL), 1011 Lausanne, Switzerland; 4 Division of Pulmonology, Ospedale Regionale di Lugano, Ente Ospedaliero Cantonale, 6900 Lugano, Switzerland; 5 Università della Svizzera Italiana (USI), 6900 Lugano, Switzerland; 6 Institute of Radiation Physics, Lausanne University Hospital, University of Lausanne (UNIL), 1011 Lausanne, Switzerland; 7 Radio-Oncology Service, Riviera-Chablais Hospital, 1847 Rennaz, Switzerland

**Keywords:** SBRT, stereotactic body radiotherapy, lung cancer, re-irradiation

## Abstract

Background/Objectives: Stereotactic body radiation therapy (SBRT) has demonstrated high local control rates for inoperable early-stage lung cancers. However, 5–15% of patients experience local relapse within the irradiated volume after treatment, with limited curative salvage options. The aim of this review is to clarify the modalities and outcomes after a second course of SBRT in patients with local relapse after a previous lung SBRT. Methods: An exhaustive literature review was conducted to identify, analyse and summarise the results of 21 main studies. Results: Local repeat lung SBRT after a first course of SBRT showed a favourable local control at 1 and 2 years, ranging from 70 to 90% and 45 to 80%, respectively. Good overall survival rates were also observed at 1 and 2 years reaching up to 95% and 85%, respectively. Toxicity was rare but could be severe, with cases of Grade 4 and 5 toxicities (≈5%). An important dose relationship was observed between re-irradiation dose levels and local control, highlighting the importance of precise dosing. The cumulative doses impacting organs at risk were similarly associated with increased radiation-induced toxicity. Central lung lesions presented a higher risk for severe side effects compared to peripheral ones. Conclusions: In conclusion, repeat lung SBRT after a first course of SBRT represents a feasible treatment option in cases of local recurrence. In order to limit severe toxicity, patients must be carefully selected, and particular attention should be given to cumulative doses to organs at risk, as well as tumour location. Thus, further investigations are still needed to refine the optimal parameters for SBRT lung re-irradiation.

## Simple Summary

Stereotactic body radiation therapy (SBRT) offers excellent local control for the treatment of inoperable early stage lung cancer. However, 5–15% of patients experience a local relapse in the irradiated volume after lung SBRT, and curative treatment options are limited. This review presents the modalities and outcomes after a second course of SBRT in patients with local relapse after a previous lung SBRT. In this review, the main studies were pooled and analysed to describe patient outcomes in terms of local control, overall survival, and toxicities.

## 1. Introduction

Lung cancer is the most lethal cancer in the world, and non-small cell lung cancer (NSCLC) represents the most common type, with an incidence of up to 80% of lung cancers [1,2]. Depending on the stage, different treatments are possible, such as surgery, chemotherapy, immunotherapy or radiotherapy. In the case of non-operable localised early-stage NSCLC, lung stereotactic radiotherapy is an established treatment [3,4,5]. Indeed, the introduction of curative lung stereotactic body radiotherapy (SBRT) for the treatment of localised lung cancer has led to a decrease in the number of untreated elderly patients, and an overall survival benefit was observed in this population, underlining the importance of screening even among the elderly [6,7]. Patients treated with surgery generally have better overall unadjusted survival, but the difference disappears when risk factors, including age and operability, are considered. SBRT could then provide surgery-equivalent survival for patients with early-stage NSCLC and is considered an efficient alternative to surgery in both operable and inoperable patients [8].

SBRT relies on high doses of radiation, delivered in a few fractions at small volumes. The aim of this technique is to optimise healthy tissue sparing thanks to its very high precision, accuracy and high gradient of dose. This technique differs from conventional radiotherapy, which usually delivers lower doses per fraction, spread over repeated sessions targeting larger volumes. One of the advantages of SBRT is its reduced toxicity by precisely targeting the areas to be treated, allowing for re-irradiation in the event of a recurrence [9]. Overall, SBRT for early NSCLC achieves high-performing local control rates of up to 98% at 3 years. Overall survival after SBRT is variable but can reach up to 95% at 3 years, depending on the studies, and serious adverse events are limited, usually occurring in less than 5% of patients [10].

Moreover, lung SBRT is a valuable option in the management of lung metastases. The median local control after SBRT treatment is remarkable, reaching 90% and 79% at one year and five years, respectively. In addition, the toxicity associated with this treatment is impressively low, with an incidence of 0.5% for acute toxicity and 1.8% for late toxicity [11]. The latter is of particular interest when treating metastatic patients who present a limited life expectancy and quality of life. Treatment-related adverse events should be a major parameter to be taken into account in the management of lung metastases.

Biologically effective dose (BED) is a calculation to compare different fractionation schemes, and it evaluates the biological effectiveness of a fractional dose regimen by taking into account the biological effects of radiation on tissues [12]. It allows the comparison of different radiotherapy fractionation regimens. The BED can be calculated as follows: BED = nd [1 + d/(α/β)], where n is the number of fractions, d is the dose per fraction (Gy) and α/β a tissue-specific ratio. For most cancerous cells, the α/β ratio is 10 Gy, while the ratio is usually 2–3 Gy for healthy tissues. It is important to note that the linear quadratic model and BED have been suggested to be incorrect when used for hypofractionation and may overestimate the effect of high fractional doses of radiation [13]. Regardless of this limitation, Onishi et al. demonstrated that a local control rate of 84% at 5 years was obtained for patients receiving lung SBRT at a dose of at least 100 Gy BED compared to 36% at 5 years in those treated with a BED < 100 Gy [14]. Therefore, a BED_10_ of at least 100 Gy is recommended as the minimum dose to be delivered to ensure optimal local control for pulmonary targets [15]. Similarly, the equivalent dose of 2 Gy (EQD2) is a dose calculation that permits the comparison of the dose efficacy of hypofractionated radiotherapy to conventional doses of radiotherapy delivered in standard fractions of 2 Gy [16].

After an initial conventional radiotherapy treatment, approximately 20–50% of patients with NSCLC and 35–50% of patients with small cell lung cancer (SCLC) experience a locoregional relapse [16]. In contrast, distant failure is the primary pattern of failure after lung SBRT, with local recurrence after SBRT affecting only 5–15% of patients [17]. In these cases, several options can be discussed, such as surgery, re-irradiation (conventional or SBRT), palliative chemotherapy or cryo-ablation [3]. The indications for salvage surgery are limited, given that this population is usually not a candidate for surgery due to comorbidities, and therefore, a second course of radiotherapy treatment remains one of the possible options [18].

In the case of re-irradiation, doses to organs at risk (OAR) and related toxicity remain the limiting factors. Typically, toxicity depends on different parameters, including the location of the lesions (central vs. peripheral lesions), the size of the lesion, the previously delivered dose and the cumulative dose. Other patient-related factors such as concomitant chemotherapies, comorbidities and age can influence toxicity outcomes [1]. Possible toxicities are typically pneumonia, fibrosis, esophagitis, chest pain/rib fracture, irradiation dermatitis or other serious/fatal events [16]. Decisions to re-irradiate local recurrences must therefore always be balanced between the expected benefits and the potential risks [19].

Re-irradiation by a second course of conventional radiotherapy is one option, but it offers poor outcomes and high rates of toxicity and should not be prioritised [20]. A more appropriate option is a second course by salvage lung SBRT. Gathering data is important to define and to specify the place of SBRT in this setting. The aim of this review is to define, describe and analyse the outcomes of patients undergoing repeat lung SBRT in patients with local relapse after a first lung SBRT. The outcome endpoints will focus on local control and overall survival, as well as adverse effects such as treatment-induced toxicity.

## 2. Method

A literature review was performed using the PubMed database. The authors searched indexed articles in the database from 2000 to August 2024. Among the keyword combinations used were (Reirradiation OR Re-irradiation OR Repeat irradiation) AND (Stereotactic Body Radiotherapy) AND (Lung Cancer OR Lung Neoplasms OR Lung metastases). Meta-analyses and large series were prioritised and described in the main text. Non-relevant articles and small series (N < 9) were excluded, as well as larger series with a minority of patients treated with SBRT (SBRT < 9). Observational studies outside the field of interest were excluded. A total of 187 articles were identified as matching the research criteria. After applying the inclusion criteria, restriction to the defined period and exclusion of small series, 21 articles were selected and analysed in terms of tumour control and/or toxicity. The literature search, selection and data extraction were independently conducted by A.S. and R.K. Any disagreements were solved with the help of a third author (M.O. or E.R.).

## 3. Results

### 3.1. Concept and Definition of Re-Irradiation

The European Society for Radiotherapy and Oncology (ESTRO) and the European Organization for Research and Treatment of Cancer (EORTC) elaborated a consensus on re-irradiation in order to distinguish different situations that can be grouped under the term of “re-irradiation” [21]. The proposals of the consensus were based on the results of an adapted Delphi process and were endorsed by the EORTC Board and the ESTRO Scientific Council.

Re-irradiation was defined as a new course of radiotherapy administered either to a previously irradiated volume (irrespective of concerns regarding toxicity) or to an area where the cumulative dose raises concerns of toxicity. Additionally, two categories, type 1 and type 2, of re-irradiations were distinguished from other situations classified as “repeat organ irradiation” and “repeat irradiation”. A type 1 re-irradiation was represented by a new radiotherapy treatment that overlaps geometrically with a previous course of radiotherapy.

**Figure 1:**
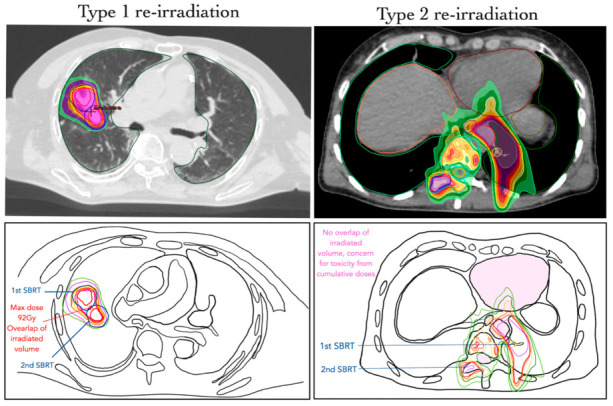
Comparative illustration between type 1 and type 2 re-irradiation. Type 1 re-irradiation involves an overlap of irradiated volume, increasing the cumulative dose to the same area, while type 2 re-irradiation is characterised by no overlap of irradiated volume, with concerns regarding toxicity from cumulative doses (in pink).

A type 2 re-irradiation was defined in the case of no geometric overlap between the irradiated areas but where there was a concern about toxicity due to cumulative doses (Figure 1). On the other hand, a “repeat organ irradiation” refers to the irradiation of an organ previously irradiated but without overlapping areas or concerns about toxicity resulting from cumulative doses. In addition, “repeat irradiation” was defined as the irradiation of two different organs without an overlapping zone and without concerns about toxicity from cumulative doses. The choice of a cut-off value for volume, overlap or time interval between treatments was rejected by ESTRO and EORTC in the re-irradiation consensus [21]. The selected articles analysed below corresponded to type 1 re-irradiation, representing cases of high doses of re-irradiation.

### 3.2. Population and Treatment Characteristics

In summary, the 21 reported studies included a number of patients ranging from 9 to 46 patients, most of them being men over 65 years old. The median age was 70 (34–94), with a male/female ratio of 62/38%. The median interval between the first and second irradiation was 18 months (1–180 months), and the median follow-up after the second irradiation was also 18 months (0.4–132 months). Central and peripheral recurrent lesions were equally distributed. The median dose administered during the first irradiation was 69 Gy (30–150 Gy), whereas it fell to 57 Gy (16–156.2 Gy) during the second irradiation. Numerous re-irradiation regimens have been published, ranging from 15 to 75 Gy in 1 to 16 fractions. An overlapping irradiation field between the two courses is usually required to be considered as a re-irradiation (corresponding to type 1 re-irradiation). Histological proof of a local relapse is usually not mandatory before re-irradiation. Dose fractionations at re-irradiation still correspond to the classical lung SBRT regimen [15]. More exhaustive data are presented in Table 1 [2,22,23,24,25,26,27,28,29,30,31,32,33,34,35,36,37,38,39,40,41,42].

A large retrospective series conducted on 31 patients by Ogawa et al. covered a period from 2004 to 2017. Concerning recurrence, 15 patients underwent histological examination, and recurrence was confirmed in 14 of them, with results showing that 10 had adenocarcinoma, 11 had squamous cell carcinoma (SCC) and 2 had non-small cell lung cancer (NSCLC). In addition to patients with lung cancers, eight patients had metastatic disease, originating from lung, breast, colon or hepatocellular carcinoma. At the first irradiation, the median tumour size was 2.2 cm and 3.2 cm at the second course of treatment. During the first treatment, patients received doses ranging from 36 Gy to 60 Gy, delivered in 2 to 10 fractions. The median time between the first and second irradiation was 18 months. During the second treatment, the dose fractionations varied between 48 and 60 Gy, delivered in 4 to 16 fractions [23].

John et al. conducted a retrospective study between 2009 and 2020 including 27 patients. Histological diagnosis showed that the primary tumour was NSCLC in 12 patients, colorectal cancer in 8 patients, 1 patient had breast cancer and 1 patient had melanoma. In the end, 11 lesions were primary recurrent tumours of the lungs, and the other 16 were recurrent metastases. Confirmation of recurrence was done by PET for 13 patients, by a chest CT for 12 patients and by biopsy for 2 patients. In the first treatment, patients received one to five fractions with a median dose of 38.5 Gy. For the second treatment, a median dose of 40 Gy in three to eight fractions was delivered. Of the 27 patients, 7 benefited from systemic therapy during their re-irradiation [24].

Another important series was published by Caivano et al., describing the results of a retrospective study of 22 patients treated between 2011 and 2016. Among these patients, some had multiple lesions, totalling 27 lesions, of which 4 were classified as central and 23 as peripheral. Twenty-one lesions were considered relapsed “in-field” (80% overlap required by the authors). During the first course of treatment, among the 22 patients, 13 had primary lung cancer, while 7 had metastases from other cancers, including ovarian, rectum, breast, uterine and oropharyngeal cancers. The diagnosis of recurrence was made by [^18^F] FDG PET/CT for 19 lesions and by CT scan for the others. At the time of recurrence, no patient had histological proof before re-irradiation. After re-irradiation, eight patients underwent chemotherapy. The equivalent dose of 2 Gy (EQD2) during the first and second treatments was 93.8 Gy and 83.3 Gy, respectively. The cumulative total median EQD2 dose was 166.7 Gy in the PTV. The median volume of the re-irradiated lesions was 30.8 cm^3^ with a median diameter of 3.7 cm [25].

Wang et al. published in 2023 a series of 17 patients. In this study, a re-irradiation setting was defined as the recurrence occurring within the field of the first tumour treatment. Fourteen patients had primary lung tumours, while three had lung metastases. The volume of the tumour at the time of the first treatment was 19.3 cm^3^, while it was 39.1 cm^3^ at the time of recurrence. Histological proof was obtained in 13 patients for the first course and in only 4 patients for the second course of treatment. The treatment regimens were 60 Gy in four fractions and 50 Gy in five fractions, with median EQD2 of 125 Gy and 100 Gy for the first and second course of radiotherapy, respectively. The cumulative median EQD2 was 225 Gy. During or after re-irradiation, six patients benefited from systemic therapy [2].

### 3.3. Patients Outcome: Local Control, Overall Survival and Prognosis Factor

The median overall survival (OS) in the 21 reviewed articles was 76% (52–95%) at 1 year, dropping to 45% (29–69%) at 2 years. The median local control (LC) was 77% (55–92%) and 65% (37–92%) at one and two years, respectively [26,27,28,32,40,42]. More exhaustive data are detailed in Table 2 [2,21,22,23,24,25,26,27,28,29,30,31,32,33,34,35,36,37,38,39,40,41,42,43].

Caivano et al. reported at 1 year and 2 years a LC of 67% and 54%, respectively, and an OS of 81% and 63%, respectively. The authors also investigated the influence of fractionation on LC (single fraction vs. multiple fractions). A notable difference was highlighted for the cumulative EQD2(10) ≥ 167 Gy vs. < 167 Gy (*p* = 0.05) for LC. No other statistically significant correlations were identified [25].

John et al. demonstrated a good LC with 70.3% at one year and 51.1% at two years. They found an OS of 78.3% at one year and 67.5% at two years. The authors of this study highlighted several factors influencing the LC and OS. They demonstrated, with high statistical significance (*p* = 0.013), that a peripheral anatomical location achieved better LC compared to central lesions. In addition, it was shown, albeit at the limit of statistical significance (*p* = 0.055), that a higher dose during re-irradiation improved local control. They also demonstrated, with strong statistical relevance (*p* = 0.005), that a higher dose was associated with better OS. Moreover, they showed that controlled disease after re-irradiation correlated with better OS. Finally, the authors established that a longer interval between the two SBRTs and the absence of metastases correlated significantly with a longer disease control rate (*p* = 0.033 and *p* = 0.023, respectively) [24].

Ogawa et al. reported a LC at 1 and 2 years of 77% and 66%, respectively. A good OS was observed with 84% at one year and 68% at two years. The authors investigated whether there was a significant relationship in terms of OS and LC between central and peripheral tumours; however, the difference was not significant (*p* = 0.75 for OS and 0.26 for LC). The authors did not identify any other significant prognostic factors [23].

In the meta-analysis published by Wang et al., the LC at one and two years was 85%. The OS at one and two years was 68% and 46%, respectively. The authors of this study also explored factors influencing the LC and OS. Regarding local control, they highlighted, with strong statistical significance (*p* < 0.001), a dose–response relationship, confirming the influence of this parameter. Specifically, 50.10 Gy in three fractions, 55.85 Gy in four fractions and 60.54 Gy in five fractions were associated with better LC rates at 2 years with a TCP of 80%. In contrast, doses of 42.04 Gy, 47.44 Gy and 53.32 Gy in five fractions had a TCP of 50%, 60% and 70%, respectively. A similar dose–response relationship was also noted in OS. For instance, doses of 43.79 Gy, 48.63 Gy and 52.55 Gy in three, four and five fractions were associated with 2-year survival rates of 70%. Conversely, lower doses of 41.62 Gy, 46.88 Gy and 52.55 Gy in five fractions produced less favourable results, with a 2-year OS of 50%, 60% and 70%, respectively (*p* < 0.01) [2].

### 3.4. Patients Outcome: Toxicity, and Prognosis Factors

Caivano et al. reported 21 Grade 1 and 2 toxicity events. In addition, they described five cases of Grade 3 toxicity, including dyspnoea, chest pain and pulmonary fibrosis, among 22 patients included in their study. No Grade 4 or 5 events were found in this study. In total, 59% of the lesions treated by Caivano et al. did not exhibit any effects related to acute or late toxicity. The authors of this study did not find a statistically significant correlation between toxicity and dose, location or volume of lesions [25].

Regarding toxicity, John et al. observed three patients with Grade 1 pneumonia, as well as one patient with Grade 2 pneumonia, among 27 patients included in their study. They did not report any Grade 3 or higher toxicity. Grade 2 pneumonia was observed in a single patient receiving immunotherapy (Nivolumab) during re-irradiation, but no association was found out [24].

Ogawa et al. identified 19 patients with Grade 1 pneumonitis among 31 patients included in their study. Four patients developed Grade 2 pneumonitis, of which three had a central tumour and one patient had a peripheral tumour. However, the authors could not demonstrate a statistically significant difference between the two groups (*p* = 0.30). Of the 31 patients, six experienced Grade 1 or 2 rib fractures. No patients developed Grade 3 or higher toxicity after the second SBRT [23].

Among the 17 patients treated by Wang et al., 11 patients experienced Grade 2 adverse toxicity events, such as pneumonitis, fibrosis, atelectasis, rib fracture and pleural effusion. In addition, only one patient with a central tumour developed Grade 3 pneumonia. The authors of this study did not report any Grade 4 or 5 toxicity. Considering the entire population of the meta-analysis by Wang et al. (N = 195), 77.36% of patients experienced Grade 1 or 2 toxicity; the most common were pneumonitis, rib fractures, chest pain and dyspnoea. Approximately 12% of patients developed Grade 3 toxicities, including pneumonitis, dyspnoea, chest parietal pain and pulmonary fibrosis. Three patients (1.4%) experienced Grade 4 toxicities, such as dermatitis, vena cava stenosis and tracheogastric fistula. Additionally, three patients with central tumours (1.42%) developed Grade 5 toxicities, particularly haemorrhage. One patient with a peripheral tumour died following gastric perforation, which was related to tumour recurrence close to the stomach and high doses treatment. A significant correlation between the location of the tumour (central vs. peripheral) and the risk of severe toxicity (*p* < 0.01) was identified. Furthermore, the cumulative dose received by OAR, particularly for central tumours, was a predictive factor for toxicity. It is worth noting that the cumulative doses on OAR, including the spinal cord, proximal bronchial tree, heart, large vessels and oesophagus, were higher for central tumours than peripheral tumours [2].

Viani et al. also investigated the influence of several factors on toxicity, including tumour location (central or peripheral), tumour size, SBRT dose administered, cumulative dose, treatment margins, chemotherapy history, patient age and comorbidities. They classified the studies into two groups according to dose delivered: EQD2 ≤ 145 Gy2 and EQD2 > 145 Gy. The results demonstrated that the cumulative dose was significantly associated with the development of severe toxicities, with a Grade 3 toxicity rate of 3% in one group compared to 15% in the other (*p* = 0.013) [1].

Concerning serious (Grade 4) adverse events, three examples of Grade 4 events were reported. First, Peulen et al. reported vena cava stenosis and trachea-gastric fistula in two cases, involving re-irradiations of central lesions [34]. Additionally one severe Grade 4 skin toxicity case was also reported by Reyngold et al. [36]. A total of 10 cases of Grade 5 lethal toxicity were also described. Kilburn et al. detailed a case of Grade 5 esophago-aortic fistula and vascular death [31]. Peulen et al. described three patients with Grade 5 bleeding [34]. Repka et al. detailed a case of Grade 5 haemoptysis [35]. Sumodhoe et al. reported two cases of Grade 5 toxicity: one alveolitis and one haemoptysis [38]. Trovo et al. described fatal Grade 5 pneumonitis, as well as Grade 5 haemoptysis [40]. These findings reveal that serious adverse events remain rare but particularly threatening in re-irradiation settings. Thus, careful attention must be paid to cumulative doses and central tumour locations. These toxicity data are summarised in Table 2 [2,22,23,24,25,26,27,28,29,30,31,32,33,34,35,36,37,38,39,40,41,42].

## 4. Discussion

Repeat lung SBRT after a first course of SBRT has shown interesting LC rates at 1 and 2 years, with maximum values of 89% and 85%, respectively. Nevertheless, these values remain lower than those observed after a first SBRT course [2,31]. One hypothesis explaining this difference in local control could be higher radioresistance after a first SBRT treatment, with higher doses required to control the recurrent tumour [39]. Indeed, if a first course of radiotherapy at an effective dose was not sufficient to achieve local control, the second course of radiotherapy should be given at a higher dose if possible. In addition, progression after a first course of radiotherapy is more likely to involve resistant tumour clones, particularly through the acquisition of epithelial-to-mesenchymal transition [43,44]. Unfortunately, re-irradiation doses are often limited by fears of toxicity, due to cumulative doses to OAR. This could partially explain the lower LC rates observed. Indeed, several factors have been identified as influencing LC—in particular, a dose–response relationship, such as EQD2(10) ≥ 167 Gy being associated with better tumour control compared to lower doses [25]. In addition, anatomical location plays an important role: peripheral lesions showed better LC compared to central lesions [24]. Good OS rates were observed at one and two years with maximum values up to 69–95% [32]. Several factors influenced the OS, and the administration of higher doses of re-irradiation was also shown to be associated with better OS [2]. These values appear comparable to the outcome after a first SBRT course. This observation reinforces the decision to deliver repeat lung SBRT when feasible and necessary [2].

Although re-irradiation with SBRT is an effective treatment option for lung tumour recurrences, toxicity is not negligible. Some authors have found a significant number of severe toxicities > Grade 2, with a few cases of fatal toxicity. Among them, some have found predictive factors for these severe toxicities, such as Viani et al., who suggested that a cumulative EQD2 dose > 145 Gy was associated with a higher risk of toxicity > Grade 2 [1]. Yang et al. found that sequential chemotherapy was linked to a higher risk of lethal pulmonary events [22]. Finally, Wang et al. found a significant correlation between central and peripheral locations and the risk of severe toxicity (*p* < 0.01), noting that higher doses were delivered to OAR (spinal cord, proximal bronchial tree, heart, great vessel and oesophagus) in central lesions [2]. It remains difficult to identify one organ at greater risk than another, mainly because of the size of the cohorts and the relatively small number of events. Among lethal toxicities, a high number of massive haemoptysis cases were noted, but either no details are given about the origin of this haemoptysis or the authors indicated that the origin was not found. Thus, the use of a more precise device for radiotherapy should be favoured. In this regard, fiducial-guided radiotherapy could help reduce doses to OAR [45,46].

In many studies, a histological confirmation of disease was provided at the time of the first SBRT, but biopsies were not repeated at the time of recurrence: some patients were then re-irradiated without pathological proof of relapse. This lack of histological confirmation of recurrence is an obstacle to the interpretation of results regarding re-irradiation and to optimal patient management. Indeed, without histological evidence, patients could be treated under the assumption of a recurrence, exposing them to potentially severe side effects unnecessarily. Moreover, it could further expose physicians to legal issues in cases of severe complications occurring in patients and without proof of relapse justifying such a challenging treatment. Histological confirmation of relapse thus appears essential to ensure the justification of aggressive therapeutic interventions while minimising the risks for patients and physicians. In the absence of possible histological proof, it remains essential to validate the indication of re-irradiation at a multidisciplinary tumour board, review imaging results with expert radiologists and nuclear medicine physicians and to discuss the benefit/risk balance with colleagues and the patient. Thus, the diagnosis of local recurrence must be based on a comprehensive set of arguments: the presence of hypermetabolism on FDG-PET/CT, sufficient radiologic certainty [47] and compatible progression kinetics.

Treating local lung recurrences after a first course of SBRT is a real challenge, as treatment options are limited. Patients who have received a first SBRT are rarely eligible for surgery. In addition, surgery and conventional radiotherapy are rarely effective in the treatment of recurrences [1]. Re-irradiation by SBRT with doses of 50 to 60 Gy in three to five fractions is feasible in selected patients with peripheral recurrence. However, for patients with centrally localised tumours, caution is necessary due to the potential risk of severe toxicity. With advances in imaging technology and irradiation techniques (such as real-time lung tumour tracking, no co-planar irradiation, etc.), it is now possible to achieve greater accuracy, allowing for the delivery of higher doses while minimising adverse toxic effects. It is also important to use the most appropriate device in order to minimise the doses to OAR. An individualised approach should determine the best technical options, such as breath hold, determination of an ITV or tracking technologies through fiducial implantation [46,48].

Additional research is needed in the future to better identify and select appropriate patients to minimise the risks of toxicity and deliver efficient SBRT treatments in cases of local lung recurrences [49]. The indication for thoracic re-irradiation should be based on a careful assessment of the risks and a rigorous selection of patients [50].

## 5. Conclusions

Published studies have revealed favourable local control rates after repeat lung SBRT, with 1-year and 2-year control rates of approximately 70–90% and 45–80%, respectively. The overall survival rates were also promising, reaching up to 95% at 1 year and 69% at 2 years. Toxicity was infrequent and mostly minor (≈15%); however, some severe cases, including Grade 4 and 5 toxicities, were reported. A notable dose–response relationship was found between the re-irradiation dose and LC. Additionally, cumulative doses to OAR were associated with an increased risk of radiation-induced toxicity, and optimal techniques with a high dose gradient should be favoured in order to minimise this parameter. Particular attention should be paid to central lesions, as they present a higher risk of severe toxicity. In conclusion, repeat lung SBRT for local recurrence is a feasible treatment option but requires careful patient selection and meticulous planning to minimise the risks of severe toxicity (≈5%). Further research is needed to establish the optimal parameters for an effective and safe treatment.

## Figures and Tables

**Table 1:** Summary of patients’ characteristics and treatment plan.

Author, Journal, Year	Study Period Study Type	Follow-Up Period (Months)	Number of Patients and Lesions	Median Age (Years)	Gender M/F (%)	Central/Peripheral Recurrence (%)	Median Time Interval with 1st RT (Months)	Median EQD2 1st RT (Gy10)	Technique of the 1st RT	Median EQD2 of Re-RT (Gy10)	Re-RT Technique and Doses
Caivano, Radiation Oncology 2018 [25]	2011–2016 Retrospective	13 (13–65)	22 pts 27 lesions	70 (47–82)	15/7 (68%/32%)	4/23 (15%/85%)	18 (6–66)	93.8 (32.5–100)	SBRT 23–60 Gy/1–30 fx	83.3 (40–126)	SBRT 30–54 Gy/1–5 fx
Ceylon, Oncol Res Treat 2017 [26]	2005–2015 Retrospective	9 (3–93)	28 pts 34 lesions	64 (48–90)	25/3 (89%/11%)	16/18 (47%/53%)	14 (4–56)	NR	1 pt: SBRT 60 Gy 27 pt: EBRT59–60 Gy	40 (20–150)	SBRT 20–60 Gy/3–9 fx
Ester, Day. of Radiosurgery and SBRT 2013 [27]	2006–2012 Retrospective	11.4 (1.6–38.3)	12 (13) pts 14 lesions	68 (45.9–86.7)	8/4 (67%/33%)	4/9 (31%/69%)	19.7 (4.7–84.7)	NR	9 pts: CFT 2 pt: SBRT 1 pt: HF	71.3 (71.3–83.3)	SBRT 45–50 Gy/5 fx
Hearn, Int J Radiation Oncol Biol Phys 2014 [28]	2004–2012 Retrospective	13.8 (5.3–43.5)	10 pts 10 lesions	72 (51–78)	5/5 (50%/50%)	2/8 (20%/80%)	14.8 (9.9–26.3)	83.3 (83.3–124.7)	SBRT 30–50 Gy/1–5 fx	83.3 (83.3–150.0)	SBRT 50–60 Gy/3–5 fx
Hong, Cancer Res Treat. 2019 [29]	2005–2016 Retrospective	17.4 (4.8–76.8)	31 pts NR lesions	64 (43.6–88.9)	27/4 (87%/13%)	NR	15.1 (4.4–56.3)	60 (45–66)	45–66 Gy 27 pt: CFT 4 pt: IMRT	NR	35–66 Gy 10 pts: SBRT 21 pt IMRT/TOMO
John, Scientific Reports 2021 [24]	2009–2020 Retrospective	17.5 (0.4–76.2)	27 pts 27 lesions	71 (34–88)	18/9 (67%/33%)	10/17 (37%/63%)	20.2 (3–89)	57	SBRT 20–85 Gy/1–5 fx	60	SBRT 19–66 Gy/1–5 FX
Kennedy, Radiother Oncol.—2020 [30]	2008–2017 Retrospective	24 (3–60)	21 pts 21 lesions	75 (59–89)	13/8 (62%/38%)	6/15 (29%/71%)	23 (7–52)	126 (83.3–126)	SBRT 50–54 Gy/3–5 Fx	83.3 (83.3–126)	SBRT 50–54 Gy/3–5 Fx
Kilburn, Radiother Oncol 2014 [31]	2001–2012 Retrospective	17	33 pts 33 lesions	66 (45–80)	19/14 (58%/42%)	17/16 (52%/48%)	18 (6–61)	SBRT: 60–150 CFRT: 42–85	10 SBRT 22.5–60 Gy/1–5 fx 23 EBRT 45–80.5 Gy/28–37 fx	SBRT 50–126 CFRT 69–70	30 pts: SBRT 20–54 Gy/1–10 fx 3 pts: EBRT 60–70 Gy/26–35 fx
Lee, Radiation Oncology 2012 [32]	2013–2018 Retrospective	28 (3.5–95.8)	20 pts 20 lesions	73 (51–85)	16/4 (80%/20%)	15/5 (75%/25%)	13.8 (2.0–51.6)	NR	6 pts: CFT 14 pts: SBRT	105.75 (72–125)	SBRT 48–60 Gy/4–6 fx
Maranzano, J Radiosurg SBRT 2015 [33]	2003- 2013 Retrospective	57 (6–132)	18 pts 29 lesions	68 (53–84)	14/4 (78%/22%)	15/14 (52%/48%)	18 (6–90)	SBRT: 32–60 Gy CFT: 60–83 Gy	11: SBRT 30–60 Gy/10–30 fx 6: CFT 40–50 Gy/5 fx	50 (31–83)	SBRT 25–50 Gy/5 fx
Ogawa, Radiat Oncol 2018 [23]	2004.2017 Retrospective	26 (5–111)	31 pts 31 lesions	78 (58–92)	24/7 (77%/23%)	9/22 (29%/71%)	18 (4–80)	93 (62.5–99.7)	SBRT 36–60 Gy/2–10 fx	85 (52–99.7)	SBRT 48–60 Gy/4–16 fx
Patel, J Radiat Oncol, 2015 [42]	2008–2011 Retrospective	NA	26 pts 29 lesions	68 (42–87)	7/19 (27%/73%)	17/12 (59%/41%)	8 (2–26)	NR	3: SBRT 26: EBRT 61 Gy	40 (16–93)	SBRT 15–50 Gy/3–5 fx
Peulen, Radiother Oncol 2011 [34]	1994–2004 Retrospective	12 (1–97)	29 pts 32 lesions	65 (18–87)	18/11 (62%/38%)	11/21 (34%/66%)	14 (5–54)	63 (30–94)	SBRT	63 (48–94)	SBRT 20–60 Gy/1–7 fx
Repka, Radiat Oncol 2017 [35]	2004–2014 Retrospective	12	20 pts 20 lesions	70 (47–90)	12/8 (60%/40%)	20/0 (100%/0%)	30.8 (2.6–93.6)	62 (58–78)	CF-EBRT 60–75 Gy/23–41 fx	50 (31–71)	SBRT 25–45 Gy/3–9 fx
Reyngold, Radiat Oncol 2013 [36]	2004–2011 Retrospective	12 (1–47)	39 pts 39 lesions	71 (41–94)	20/19 (51%/49%)	NR	37 (1–180)	NR	13 pts: CFT 12 pts: 3 dCRT 14: IMRT	38 (23–98)	SBRT 42–60 Gy/1–5 fx
sood, Clinical Lung Cancer 2021 [37]	2009–2017 Retrospective	18 (3–67)	20 pts 21 lesions	73 (58–89)	7/13 (35%/65%)	21/0 (100%/0%)	14 (4–100)	NR	EBRT 50–70 Gy SBRT 50–70 Gy	53 (28–59)	hSBRT 40–70 Gy/10 fx
Sumodhee, BMC cancer 2019 [38]	2007–2015 Retrospective	47 (1–77)	46 pts 46 lesions	66 (44–83)	35/11 (76%/24%)	24/22 (52%/48%)	23 (6–102)	66 (32–75)	CFT 44–70 Gy/19–38 fx	130 (66–156.2)	SBRT 40–75 Gy/3–5 fx
Trakul, J Thorac Oncol 2012 [39]	2004–2010 Retrospective	15 (5–65)	15 pts 17 lesions	66 (49–92)	7/10 (41%/59%)	6/11 (35%/65%)	16 (5–80)	72 (50–94)	4 pts: SBRT 20–50 Gy/1–4 Fx 3 pts: IMRT 10 pts: 3 dCFT	72 (50–94) or 66 (50–94)	SBRT 20–50 Gy/1–5 Fx
Trovo, Int J Radiation Oncol Biol Phys 2014 [40]	NA Retrospective	18 (4–57)	17 pts 17 lesions	66 (40–88)	14/3 (82%/18%)	17/0 (100%/0%)	18 (1–60)	48–65	50–70 Gy/20–30 fx 11 CRT 6 IMRT	37 (37–40)	SBRT 30 Gy/5–6 Fx
Valakh, J Can Res Ther 2013 [41]	2006–2011 Retrospective	22 (4–40)	9 pts 9 lesions	74 (59–83)	0/9 (0%/100%)	NR	11 (1–25)	110 (50–150)	SBRT 30–60 Gy/3–5 Fx	110 (50–150)	SBRT 30–60 Gy/3–5 fx
Wang, Radiotherapy and Oncology 2023 [2]	NA Retrospective	21.9 (5.9–71.0)	17 pts 17 lesions	73 (54–87)	13/4 (76%/24%)	5/12 (29%/71%)	15.7 (9.1–83.5)	125 (79.3–150)	SBRT 50–60 Gy/3–8 Fx	100 (72.0–150)	SBRT 48–60 Gy/3–6 fx

NR: not reached, NA: not applicable.

**Table 2:** Summary of the overall survival, local control and toxicity.

Author, Journal, Year	Overall Survival		Local Control		Toxicity
	1 Year	2 Years	1 Year	2 Years	
Caivano, Radiation Oncology 2018 [25]	81%	63%	67%	54%	G1–2 dyspnea: 10 pts; G2 chest pain: 1 pt; G1 laryngeal haemorrhage: 1 pt; G2 peripheral sensory neuropathy: 1 pt; G1 cough: 1 pt; G1 productive cough: 1 pt; G1–2 pulmonary fibrosis: 2 pts; G1 rib fracture: 1 pt; G2 pleural effusion: 1 pt; G2 gastroesophageal reflux disease: 1 pt; G2 soft tissue necrosis: 1 pt. G3 dyspnea: 2 pts; G3 chest pain: 1 pt; G3 pulmonary fibrosis: 2 pts.
Ceylon, Oncol Res Treat 2017 [26]	71%	42%	69%	37%	7 pts: G1–2 cough and dyspnea without need for O2, 1 pt: G2 radiation pneumonia, 1 pt: death 6 months
Ester, Day. of Radiosurgery and SBRT 2013 [27]	80%	36%	92%	92%	1 pt: G2 lobar atelectasis, 1 pt: G3 pneumonia
Hearn, Int J Radiation Oncol Biol Phys 2014 [28]	NR	NR	60% (median of 9.9 months)		5 pt: G1–2 chest wall pain, 3 pt: G1–2 fatigue
Hong, Cancer Res Treat. 2019 [29]	76.8%	39.4%	60.2%	43.7%	4 pt: G1 Acute esophagitis, 2 pt: G1 acute pericarditis, 13 pt: G1–2 Symptoms, acute pulmonary, 5 pt: G1–2 acute chest wall pain, 2 pt: G1 acute radiation dermatitis 2 pt: G1–2 Chronic esophagitis, 4 pt: G1–3 chronic pericarditis, 15 pt: G1–2 Symptoms, chronic pulmonary, 2 pt: G1–2 acute chest wall pain, 2 pt
John, Scientific Reports 2021 [24]	78.3%	67.5%	70.3%	51.1	3 pts: G1 pneumonia, 1 pt: G2 pneumonia
Kennedy, Radiother Oncol. 2020 [30]	~83%	68%	NR	81%	2 pts: G2 pneumonia, 4 pts: G2 chest wall pain
Kilburn, Radiother Oncol 2014 [31]	76%	45%	~89%	~67%	no grade esophagitis: 1 pt, G2–3 chest wall pain: 6 pts, G2–3 pneumonia: 3 pts, G5 esophago-aortic fistula: 1 pt, vascular lesion with death: 1 pt
Lee, Radiation Oncology 2012 [32]	95%	69%	73.9%	63.3%	1 pt: G1 acute cough, 3 pts: G2 acute cough, 1 pt: G1 acute dyspnea, 3 pts G2 acute dyspnea, 2 pt: G1 acute chest pain, 1 pts G2 acute chest wall pain, 1 pt: G2 chronic cough, 1 pt: G3 chronic dyspnea, 1 pt: G2 chronic chest wall pain
Maranzano, J Radiosurg SBRT 2015 [33]	~87%	~65%	82%	66%	1 pts: G1 dysphagia, 1 pts: G1 thoracic pain, 1 pts: G1 asymptomatic pneumonia
Ogawa, Radiat Oncol 2018 [22,23]	84%	68.4%	77%	66%	G1 pneumonia 19 pts; G2 pneumonia: 4 pts; G1–2 rib fracture: 6 pts;
Patel, J Radiat Oncol, 2015 [42]	52%	37%	78%	65%	G1–2 cough: 3 pts, G2 pneumonia: 1 pt, G1 esophagitis: 4 pts, G1 skin involvement: 1 pt, G1 fatigue: 5 pts, G1 dyspnea: 2 pts
Peulen, Radiother Oncol 2011 [34]	59%	43%	NR	NR	G1–2 atelectasis: 8 pts; G1–2 cough: 10 pts; G1–2 dyspnea 7 pts; G2 pneumonitis: 3 pts; G1–2 pleural effusion: 6 pts; G1–2 pulmonary fibrosis: 11 pts; G1 fracture: 1 pt; G2 dermatitis: 1 pt; G1–2 hyperpigmentation: 2 pts; G1–2 pain: 6 pts; G2 mucus production: 1 pt. G3 cough: 3 pts (central); G3 dyspnea 1 pt (central) and 3 pts (peripheral); G3 pneumonitis: 1 pt (central); G3 Airway Stenosis: 1 pt (Central); G5 haemorrhage: 3 pts (middle); G3 pleural effusion: 1 pt (peripheral); G2 dermatitis: 1 pt (peripheral); G3 pain: 1 pt (central); G4 superior stenosis of the vena cava: 1 pt (central); and G4 fistula between the trachea and the gastric tube: 1 pt (central).
Repka, Radiat Oncol 2017 [35]	45% (<40 Gy) 77% (>40 gy)	NR	66% (>40 Gy) 0% (<40 Gy)	NR	G2 pneumonia: 2 pts, G2–3 Recurrent laryngeal nerve involvement: 2 pts, G5 hemoptysis: 1 pt
Reyngold, Radiat Oncol 2013 [36]	~70%	45%	77%	65%	G2–3 Pulmonary involvement: 9 pts, G2–3 chest wall pain: 7 pts, G2 fatigue: 6 pts, G2-Water and soft tissue damage 1 pt, G4-skin/soft tissue involvement 1 pt
sood, Clinical Lung Cancer 2021 [37]	68%	37%	83%	40%	G2 pneumonia: 6 pts, G3 pneumonia: 1 pt, Fatal hemoptysis: 2 pts
Sumodhee, BMC cancer 2019 [38]	~65%	49%	NR	NR	G1–2 asthenia: 6 pts, G1–2 alveolitis: 6 pts, G2 pneumonia: 1 pt, G2 dysphonia: 1 pt, G1, cough: 2 pts, G1–2 esophagitis: 2 pts, G1 colors: 2 pts, G2, rib fracture: 1 pt, G3 breast cancer: 1 pt, G5 alveolitis: 1 pt, G5 hemoptysis: 1 pt
Trakul, J Thorac Oncol 2012 [39]	~80%	~35%	~65%	~49%	G2 chest wall pain: 1 pt, G2 ipsilateral vocal cord paralysis: 1 pt
Trovo, Int J Radiation Oncol Biol Phys 2014 [40]	59%	29%	86%	NR	G3 radiation pneumonia: 4 pts, G5 fatal pneumonia: 1 pt, G5 hemoptysis: 1 pt, G2 Esophagitis: 1 pt,
Valakh, J Can Res Ther 2013 [41]	100%	68%	NR	NR	G2 Pneumonia: 2 pts, G2 Dyspnea: 1 pt, G2 Chest wall pain: 2 pts, G2 brachial plexoplakia: 1 pt, G3 dyspnea: 2 pts, G3 Chest wall pain: 1 pt
Wang, Radiotherapy and Oncology 2023 [2]	68.%	46.8%	85.6%	85.6%	G1 Pneumonia: 3 pts; G2 pneumonia: 1 pt; G2 rib fracture: 1 pt; G1 pulmonary fibrosis: 2 pts; G1 atelectasis 2 pts; G2 pleural effusion: 2 pts G3 pneumonia: 1 pt

NR: not reached, NA: not applicable.

## Data Availability

Dataset is available on request from the authors.
